# Grasping Objects with Environmentally Induced Position Uncertainty

**DOI:** 10.1371/journal.pcbi.1000538

**Published:** 2009-10-16

**Authors:** Vassilios N. Christopoulos, Paul R. Schrater

**Affiliations:** 1 Department of Computer Science and Engineering, University of Minnesota, Twin Cities, Minneapolis, Minnesota, United States of America; 2 Department of Psychology, University of Minnesota, Twin Cities, Minneapolis, Minnesota, United States of America; Northwestern University, United States of America

## Abstract

Due to noisy motor commands and imprecise and ambiguous sensory information, there is often substantial uncertainty about the relative location between our body and objects in the environment. Little is known about how well people manage and compensate for this uncertainty in purposive movement tasks like grasping. Grasping objects requires reach trajectories to generate object-fingers contacts that permit stable lifting. For objects with position uncertainty, some trajectories are more efficient than others in terms of the probability of producing stable grasps. We hypothesize that people attempt to generate efficient grasp trajectories that produce stable grasps at first contact without requiring post-contact adjustments. We tested this hypothesis by comparing human uncertainty compensation in grasping objects against optimal predictions. Participants grasped and lifted a cylindrical object with position uncertainty, introduced by moving the cylinder with a robotic arm over a sequence of 5 positions sampled from a strongly oriented 2D Gaussian distribution. Preceding each reach, vision of the object was removed for the remainder of the trial and the cylinder was moved one additional time. In accord with optimal predictions, we found that people compensate by aligning the approach direction with covariance angle to maintain grasp efficiency. This compensation results in higher probability to achieve stable grasps at first contact than non-compensation strategies in grasping objects with directional position uncertainty, and the results provide the first demonstration that humans compensate for uncertainty in a complex purposive task.

## Author Summary

Optimal sensorimotor control models actions as decisions that maximize the desirableness of outcomes, where the desirableness is captured by an expected cost or utility to each action sequence. These models provide explanations for many aspects of our ability to compensate for uncertainty, but they have not been applied to understanding purposive movements—movements involving the application of forces to change the relative position of objects and the actor in the environment. Using time efficiency as a natural cost function, we present a statistical optimal control analysis of uncertainty compensation strategies in a purposive movement task, grasping an object with directional position uncertainty. In accord with the predictions of the analysis, the experimental results showed that people compensate for uncertainty by adopting grasp strategies that increase the chance to produce a stable grasp at first contact. Our findings suggest that visuomotor system plans for uncertainty even in complex purposive movements.

## Introduction

Optimal sensorimotor control models actions as decisions that maximize the desirableness of outcomes, where the desirableness is captured by an expected cost or utility to each action sequence. These models provide explanations for many aspects of our ability to compensate for uncertainty [1]–[3]. In particular, humans are near optimal at integrating sensory information with internal models of motor actions to produce estimates of world states and action consequences [4]–[7]. Moreover, people maintain and update estimates of their uncertainty, and use this information to improve task performance and economic gain [8]–[13]. The vast majority of research on optimal visuomotor control involves point-to-point movements. However, these studies have neglected normal purposive movements involving the application of forces to objects in our environment, with the intent of changing either the object's motion, as in grasping, or our own motion, as in walking. Planning for such movements requires anticipating the effects of object-body contact on subsequent dynamics. Due to the complexity of anticipating the effects of applied forces to object motion, it is significantly more challenging to adapt the optimal sensorimotor control framework to problems like grasp planning, and it is much less clear that the visuomotor system will have models complex enough to allow for optimal control strategies.

In grasp planning, fingers must be targeted toward points on the object's surface that will allow the application of forces sufficient for lifting and dexterously manipulating the object. In particular, the finger-object contacts should permit forces that are capable of stably lifting the objects and counterbalance external forces and torques exerted on the object – termed *force-closure grasping* [14],[15].

Once people place their fingers on contact points supporting force-closure, they can begin to lift the object. Hence, the duration of a grasping task depends on the time to produce force-closure grasping, and this time is minimized by movement trajectories that produce force-closure at first contact. Failure to satisfy force-closure conditions at first contact requires subsequent adjustments to rearrange the contact points – a process that requires extra time and effort. Little is known about how people recognize contact points supporting force-closure or how this process is affected by uncertainty.

**Figure 1:**
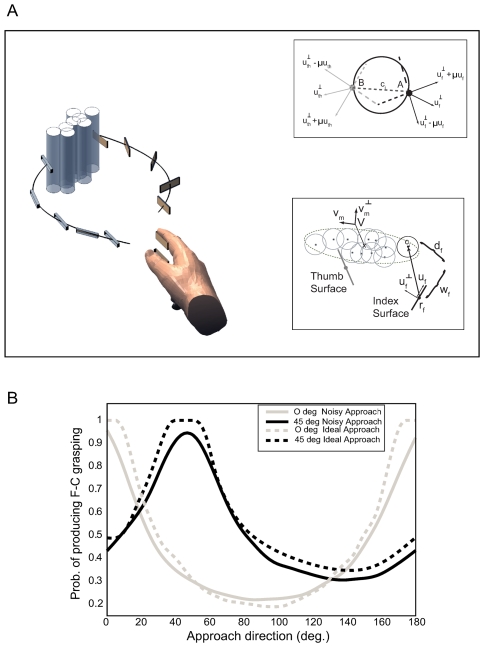
Graphical illustration of grasp analysis with directional position uncertainty. (A) The critical aspect of grasping an object with position uncertainty is the control of the contact surfaces of the index finger and thumb (rectangular patches). These surfaces must be moved along paths that will make appropriate contact with the object at any of its possible locations (gray transparent cylinders). Appropriate contact involves the concept of force-closure (see materials and methods). *Upper inset*: Force-closure grasping representation. Assume that a reach trajectory results in two contact points of the index finger (A) and the thumb (B) on the surface of the cylinder **c_i_**. This trajectory produces force-closure grasping because the line segment (AB) is located between the two friction cones defined by the contact points A and B. Based on the Coulomb's law, two contact points produce force-closure when the component of the contact forces at these points in the direction of the surface normals (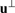) exceeds the coefficient of friction *μ* times the tangential components. The friction cones are determined by the vector 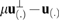 and 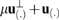, where (.) corresponds to subscript *th* and *f* for the thumb and the index finger, respectively. Note that **u** refers to the surface tangents. *Lower inset*: Graphical representation of the fingers' contact surface approach for grasping a cylindrical object with directional position uncertainty. The thumb and the index finger contact surfaces are displayed as line segments with local position **r** and normals **u**. For visualization reasons, we present only the characteristics of the index finger, whereas the characteristics of the thumb is similar to the index finger, but with the subscript *th*. The gray circles describe the possible cylinder locations based on the object's position distribution, which is illustrated as ellipse with center **x** and major and minor axes 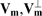, respectively. Given a possible cylinder location **c_i_**, the reach trajectory will produce force-closure if the line segment defined by contact points of the thumb and the index finger surface on the cylinder surface, is between the two friction cones at the two contact points. Note that (*w*, *d*) corresponds to the local contact coordinates of the index finger (with subscript *f*) and thumb (with subscript *th*) (see materials and methods section). (B) Effects of approach direction in the probability of producing force-closure for ideal (dashed lines) and noisy (solid lines) approaches for 0 deg (gray) and 45 deg (black) covariance orientations. Noisy approaches were generated by adding noise to both approach direction (variance = 4.5 deg^2^) and fingers orientation (variance = 2.5 deg^2^).

The purpose of our work is to study uncertainty compensation in grasping and compare human performance against normative predictions. An illustration of precision grasping objects with position uncertainty is presented in Fig. 1A. If the position of the cylinder is precisely known, all the movement trajectories will produce force-closure grasping at first contact. However, if the cylinder position is distributed along a strongly oriented 2D Gaussian distribution, some of the movement trajectories are more efficient in terms of force-closure grasping than others. The probability of achieving force-closure grasping at first contact as a function of index finger/thumb approach is presented in Fig. 1B (see Text S1 and results section for more details). The grasp trajectory that produces force-closure grasping irrespective of the cylinder position involves index finger and thumb approaching the distribution center in opposite directions along the axis of maximal uncertainty. This predicts that people should reorient the approach direction of their hand to grasp the cylinder along the direction of maximal uncertainty.

We test what, if any, changes in grasping strategy occur as compensations for object location uncertainty. Using a robotic arm to generate oriented distributions of cylindrical object locations, we investigate whether people adopt grasping strategies that minimize the impact of uncertainty on grasp success in terms of force-closure grasping at first contact.

## Results

**Figure 2:**
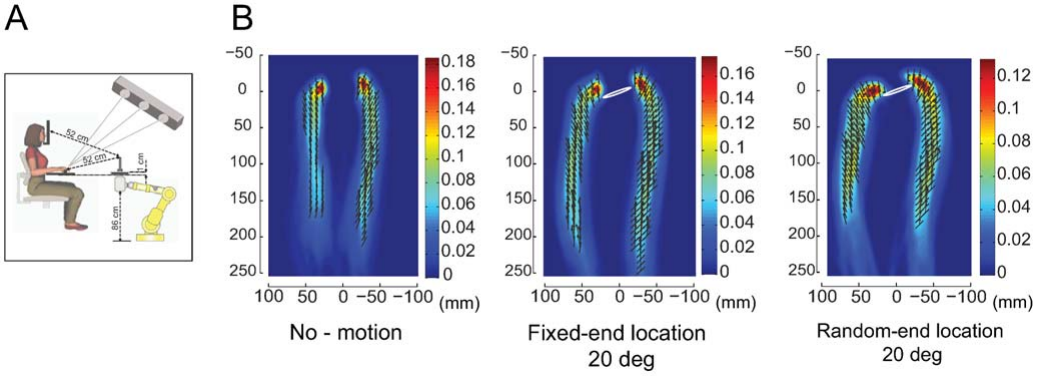
Experimental apparatus and trajectory characteristics. **(A)** Diagram illustrating the experimental apparatus. **(B)** Examples of trajectory characteristics for the three conditions, left: no motion, middle: fixed-end location and right: random-end location. The reach strategy in each condition is revealed by an analysis of the average orientation (black line segments) and velocity (arrows) of the finger's and thumb's contact surfaces at each location along the trajectories. A superimposed density map shows the probability of a trajectory passing through each spatial location, where blue and red indicate zero and high probability, respectively.

A schematic representation of the apparatus is shown in Fig. 2A. Participants were instructed to reach rapidly with the right hand to precision grasp and lift a cylindrical object mounted on a robotic arm, in 3 conditions that varied in the amount of position uncertainty. In one condition the cylinder was stationary throughout the reach (***no motion condition***). In the other two, the object visibly moved through a series of randomly drawn positions. After the object was occluded, it either moved to a new random location (***random-end location condition***) or to a fixed location (***fixed-end location condition***). The chance that the reach trajectory will end with the fingers making object contact at points permitting force-closure depends on the path the index finger and thumb take to the object. The critical part of the trajectory occurs near the end of the movement, when the fingers approach possible object locations.

Trajectory characteristics are illustrated for a single participant on the no-motion (left), fixed-end location (middle) and random-end location (right) conditions in Fig. 2B, which shows statistics on the set of trajectories in each condition. The three panels display the frequency that trajectories passed through each spatial location as a color map, where blue indicates probabilities near zero and red indicates high probability regions. The position distribution of the cylinder is illustrated by the white ellipse. In addition, we computed the average velocity and orientation of the contact surfaces of both index finger and thumb at each spatial location. The results illustrate the highly stereotyped trajectories in our experiment, with an initial forward transport phase that brings the index finger and thumb near the object, followed by an approach period in which both digits move more slowly and mainly in a direction perpendicular to the contact surface of the fingers. Note that this perpendicular approach is a critical component for producing force-closure grasping at first contact and that it is much more prominent in the random-end location condition. In addition, there are significant differences on the grip aperture profile across the 3 conditions. While grip aperture remained narrow for most of the reach trajectory in the no-motion condition, it widened near the beginning of the trajectory in the random-end location and fixed-end location conditions. Interestingly, grip aperture is larger in the fixed-end location condition than the no-motion condition, suggesting that object motion induces uncertainty even for an object felt to be in exactly the same position on every trial.

We investigated whether participants modified their index finger/thumb approach direction with the orientation of the object position distribution. We predicted a change in approach direction from an analysis of the conditions for force-closure at first contact. Force-closure occurs when the fingers make contact on the object at locations that permit the application of forces in the direction of both the surface normals and surface tangents that can potentially cage the object. A necessary condition for force-closure is that the required tangential forces are less than the force applied to the surface normals, scaled by the coefficient of friction. Geometrically, this relation produces friction cones at the contact points of the thumb and the index finger with cylinder, whose boundaries are determined by the surface tangent and normal vectors of both fingers (Fig. 1A upper inset). In the presence of object location uncertainty, approach direction affects the ability to achieve force-closure, as illustrated in Fig. 1. Particularly, Fig. 1A shows a graphical representation of the trajectory of the index finger and thumb contact surfaces (rectangular patches) to an array of possible object locations, displayed as transparent cylinders. The relevant geometry for computing force-closure for a possible cylinder location is presented in the lower inset. The center of the distribution of cylinder locations is shown as **x**, the principal axis of uncertainty as **v** ***_m_***, and the thumb and index finger contact surfaces are shown as line segments, with local position **r** and direction **u**. For each possible cylinder location **c**_i_, force-closure is only possible if the cylinder surface is in the approach path for both digits (w_f_ is less than the finger surface width) and the angle between the index finger and thumb is sufficiently large.

On the basis of this analysis, the choice of a grasp strategy can be turned into a statistical decision problem, where the objective is to select an approach that maximizes the probability of force-closure at first contact (see materials and methods section). The analysis shows that the optimal approach direction is aligned with the major axis of the covariance of cylinder locations. For a given trajectory, we can compute the probability of force-closure by determining the proportion of sample cylinder locations that satisfy the force-closure conditions. Fig. 1B shows the theoretical impact of varying approach direction on the probability of force-closure for ideal approaches (dashed lines) and noisy approaches (solid lines) generated with random perturbations added to both the approach direction (variance = 4.5 deg^2^) and the finger surface orientations (variance = 2.5 deg^2^). The results are shown for two orientations of object location uncertainty, 0 (gray) and 45 deg (black). The principal effect of additional variability is to narrow the range of approaches that produce high force-closure probabilities.

**Figure 3:**
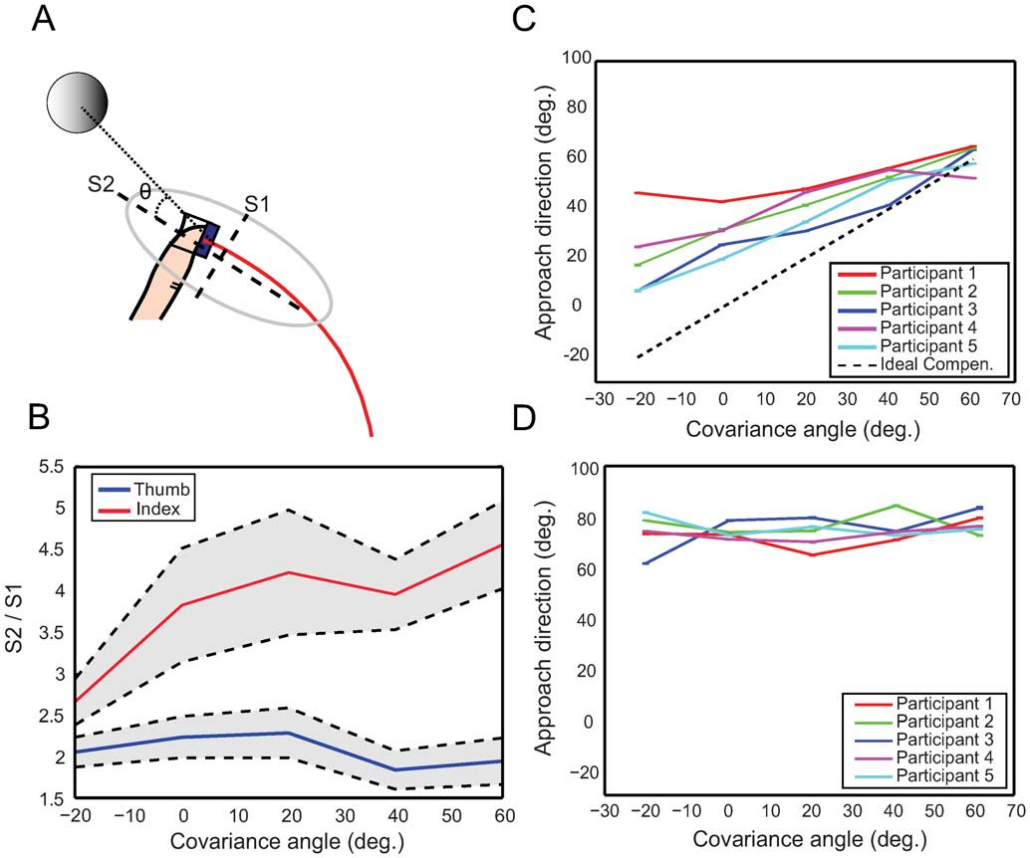
Approach direction vs. covariance angle for the “random-end location” and “fixed-end location” conditions. **(A)** Diagram illustrating the approach direction computation and definition of the covariance angle. The gray solid circle represents the cylinder location, the red line represents the average trajectory, and ellipse represents the covariation of the spatial distribution of finger locations across trajectories for the last 10 time steps preceding contact. S1 and S2 correspond to the minor and major axes of the trajectory covariance ellipse, and approach angle θ is the angle between *S2* and the x-axis (dotted line). **(B)** Average ratio *S2/S1* across participants for each covariance angle of the major and minor axes of the trajectory covariance for the thumb (blue line) and the index finger (red line), with standard errors shown in gray. **(C)** Approach direction for each covariance angle and participant in the random-end location condition. Error bars are ±1 standard error. The black discontinuous line shows ideal compensation. All participants less than fully compensate with slopes (0.26, 0.4, 0.58, 0.65, 0.67) uniformly less than the ideal of 1. **(D)** Same as C for the fixed-end location condition.

**Approach direction compensation:** If participants exhibit the predicted compensation, we should observe approach direction vary to align with the axis of maximal uncertainty. We estimated the planned approach direction for each participant and condition by computing the covariance of sensor positions across trajectories, illustrated in Fig. 3A. The direction of approach was extracted from the principle axis of the covariance spatial distribution of finger locations across the set of trajectories gathered over the ten time steps closest to the average first contact (see materials and methods section). To insure the covariance estimates were based on compatible trajectory points we restricted the analysis to a cluster of trajectories with similar temporal characteristics, comprising about 80% of the trajectories in an experimental condition. This measure captures how trajectories are spatially constrained near contact, with the main axis providing a measure of the direction of the constraint, and the ratio between major and minor axes of the covariance provides a measure of the importance of the constraint. The major/minor axis ratio, averaged across participants for each covariance angle is plotted in Fig. 3B, shows that the approach direction of the index finger is significantly more constrained than the thumb. Constraint differences in the index finger and thumb may be explained by differences in the timing of first contact – the index finger typically contacted before the thumb. Once contact is made, there are additional forces on the hand affecting the trajectories and additional information about the object's location that may influence the subsequent trajectory path. In fact, we found that trajectories in the thumb frequently exhibited direction changes after first contact by the index finger. These changes may arise because the index finger is longer and has a degree of freedom more than the thumb, making adjustments to the location and orientation easier.

Because the data suggest that locus of control is the index finger, we focused our analysis of approach direction on the index finger, shown in Fig. 3C. In the random-end location condition, where the final object location varied unpredictably, approach direction is significantly related to the object uncertainty direction (R-square>0.8038, F_[0.05;1,3]_>12.2905 and P<0.0393), and scales almost linearly for all participants. However, all participants' slopes were less than predicted by ideal compensation (shown by the black discontinuous line). In contrast, for the fixed-end location condition, where the final position was fixed across trials, the approach direction was near constant (Fig. 3D).

### Does compensation increase the probability of force-closure grasping?

**Figure 4:**
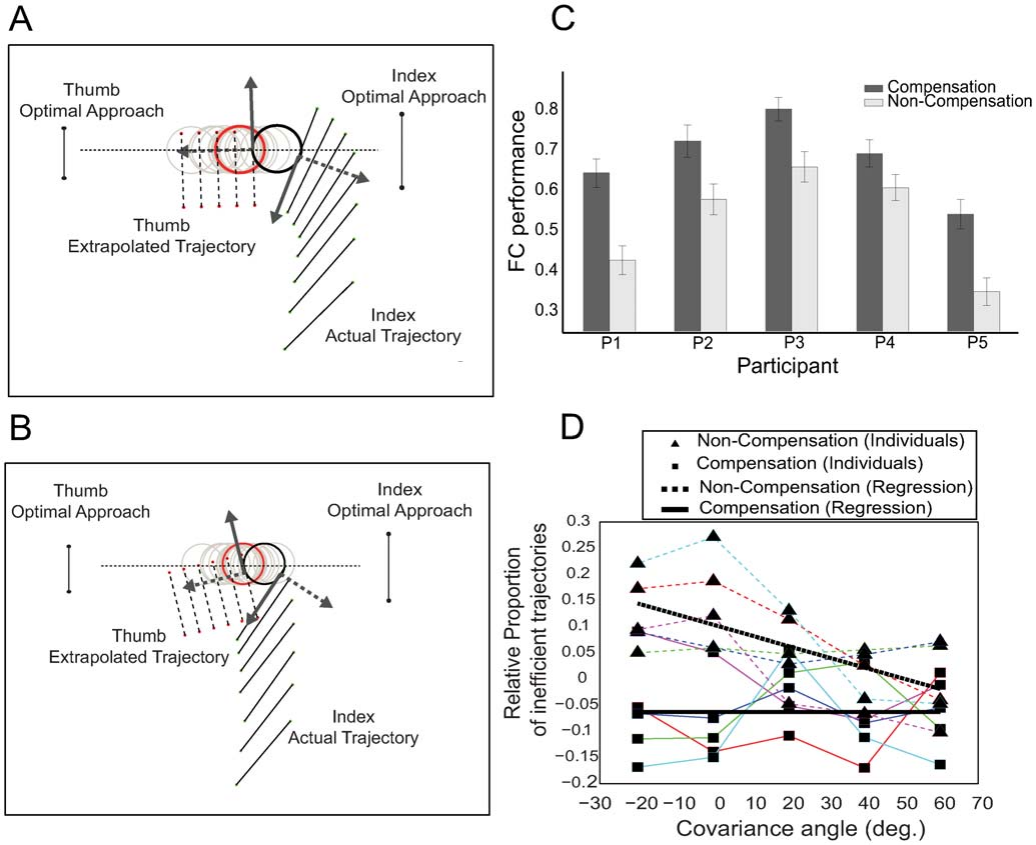
Force-closure performance evaluation. **(A)** Illustration of the analysis for computing force-closure for a given reach trajectory. Gray circles represent possible cylinder locations sampled from a covariance matrix with major axis along the dotted line, and the red circle shows the actual cylinder location. Index finger and thumb contact surface locations are illustrated by a time series of line segments. The black circle shows index finger contact with a sampled cylinder location. Once the index finger contacts a possible location, the thumb is extrapolated to assess whether the trajectory would satisfy the conditions for force-closure. This reach trajectory produces force-closure grasping. **(B)** Illustration of a trajectory with force-closure failure for most of the sample locations. **(C)** Probability of producing force-closure is shown for each participant adjacent to a simulated non-compensation strategy. **(D)** Relative proportion of *inefficient trajectories* – trajectories that produce force-closure for less than 20% of sample location. Square and triangle points represent estimates of the proportion of inefficient trajectories vs. covariance angle for each participant (each color corresponds to a particular participant same as Figs. 3c and 3d). Note that ±1 standard errors of these estimates are so small that are not visible as error bars on the figure. Dashed and solid lines show the regression results across participants.

To quantify how much the change in approach direction observed in the random-end location condition affected grasp efficiency, we computed the probability of force-closure for each participant and covariance angle. We estimated the force-closure probability for each trajectory by determining the proportion of sampled object locations that would satisfy the force-closure conditions at first contact, and averaged across trajectories (see materials and methods section). An ideal grasp strategy with no approach noise has a probability of one. To illustrate the computation, two different trajectories of a single participant with high and low probability of force-closure are shown in Figs. 4A and 4B, respectively. Sample cylinder locations are shown in gray, red describes the actual cylinder position, and the approach of the index finger and thumb contact surfaces are illustrated by a time series of line segments. In Fig. 4A the index finger makes contact with a sample cylinder location (black), which given the approach of the thumb, satisfies the criteria for force-closure. In fact, this trajectory will produce force-closure for almost all the cylinder locations generated from this distribution (0 deg orientation). In contrast, the trajectory in Fig. 4B fails to produce force-closure for the location shown in black, and for most of the other sample locations.

The probability of force-closure provides a measure of the benefits of modifying approach direction with uncertainty. Fig. 4C shows the results (averaged across covariance angles) from the random-end location condition. To provide a baseline measure of performance, we compared these results to a simulated non-compensation strategy, in which we compute the probability of force-closure for each covariance angle using the trajectories from the no-motion condition (see materials and methods section). The difference between these probabilities is a measure of the benefits achieved by approach compensation.

We can also gain insight into the benefits of compensation by focusing on the set of trajectories with low performance. We identified the set of trajectories that produced force-closure for less than 20% of sample locations (values between 20% and 50% produced similar results). Because these trajectories will require post-contact adjustments in fingers positions before lift occurs for the majority of cases, we call this measure the *proportion of inefficient trajectories*. The results from this analysis were compared with the results from the hypothetical scenario in which people do not compensate (simulated non-compensation strategy) across all covariance angles. Note that participants showed large differences in this measure but similar trends. To make these trends easier to visualize, we subtracted the mean across covariance angles of this measure from each participant's data. The results are shown in Fig. 4D. On average there were fewer inefficient trajectories in the random-end condition than the simulated non-compensation strategy. The dashed and the solid lines summarize the regression results across participants for both the simulated non-compensation strategy and the random-end location condition. In accord with expectations, a non-compensation strategy produces inefficiency curves that significantly vary as a function of covariance angle (R-square = 0.9309, Slope = −0.0022, F_[0.05; 1,3]_ = 40.4193 and P = 0.0079). In contrast, the inefficiency curves in the random-end location condition shows that by modifying approach direction, participants were able to maintain a low inefficiency rate for all covariance angles (R-square = 0.0015, Slope = 3.4900e-005, F_[0.05; 1,3]_ = 0.0044 and P = 0.9513). Moreover, a test of the two regression results shows these trends are different (ANCOVA, F_[0.05; 1,6]_ = 11.95 and P = 0.0135).

### Effects of position uncertainty on grip aperture profile

**Figure 5:**
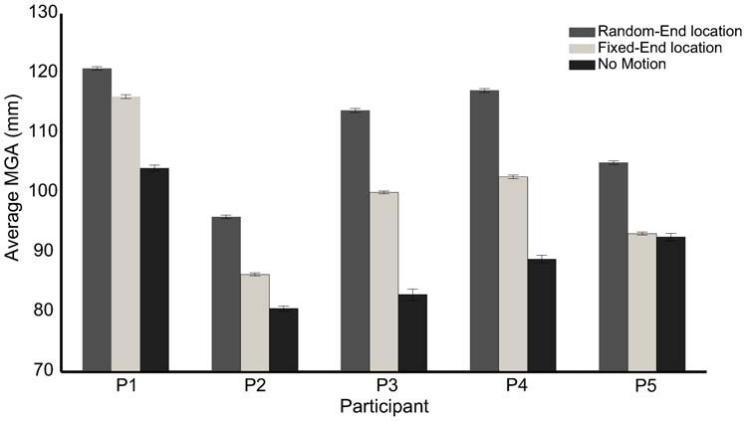
Maximum Grip Aperture (MGA). MGA averaged across trajectories and covariance angle for each condition and participants.

We also tested whether people modify their grip aperture profile as a function of condition and covariance orientation, by computing the mean value of the maximum grip aperture (MGA) across trials and regressed the results against covariance angles. For all conditions, we found no significant variation of MGA magnitude or time with covariance angle. However, there were significant differences across conditions for all participants (one-way ANOVA, F_[0.05; 1, 598]_>106.4595 and P-value<0.001), excluding Participant 5, shown in Fig. 5. Interestingly, almost all participants increased their gripwidth on the “fixed-end location” condition in response to the observed object motion, despite the fact that the final object position is both visible and always the same.

## Discussion

We have adopted principles from Statistical Decision Theory [16] to account for human behavior in a purposive movement task: grasping objects with position uncertainty. Previous studies have applied Statistical Decision Theory to model reach behavior, providing evidence that the sensorimotor system's computation can be modeled as Bayes optimal, incorporating proprioceptive and environmental information to minimize the effect of uncertainty on task performance [3], [9]–[11], [17]–[18]. To simplify modeling and the assessment of optimality, these studies have focused on artificial environments and simple tasks involving point-to-point reaching movements. It is quite challenging to extend the optimality approach to the study of purposive movements. The goals and the consequences of such movements are determined by the application of forces and the effects of noise and uncertainty, but movement plans need to be expressed in terms of limb motions. Previous studies involving purposive movement tasks have provided evidence that people modulate their grasping strategies when they can predict changes in objects' intrinsic characteristics, such as center of mass [19]–[22], surface shape [23], texture [24], and weight [25]. Particularly, the sensorimotor system uses information acquired from previous manipulations of the same object to select digit contact points and the forces required for object manipulation. However none of these studies provide models that can explain the anticipatory motor behavior that participants adopted in grasping. One of the novelties of the current work is that we used normative predictions to evaluate the benefits of uncertainty compensation, based on the hypothesis that participants attempt to minimize overall the grasp time and use this idea to construct a natural cost function on grasp approach based on the concept of force-closure grasping at first contact. We showed how the approach phase of a reach trajectory determines the time efficiency with which an object can be grasped and lifted, and generated testable predictions: participants should modify approach to allow grasp closure along the direction of maximal uncertainty and increase peak gripwidth (see Text S1). We observed both kinds of compensation strategies across conditions; a gripwidth change without approach modification in the fixed-end position condition, but both gripwidth and approach changes in the random-end position condition. Previous studies have also observed gripwidth compensation for uncertainty. In particular, MGA increases without visual information [26] and people modify their MGA with the amount of visual uncertainty about object position [27]–[28]. However, MGA did not scale with the orientation of the cylinder position distribution.

Note that modifying the approach direction requires extra energy to rotate the hand, which means that the advantages of compensation in terms of efficiency must outweigh these costs. Non-compensation strategies increase the chance of not producing force-closure at first contact, which must be corrected by time-consuming and metabolically costly finger repositioning after contact. Finger repositioning occurs in a sensory feedback loop that takes time. Contact locations and forces must be sensed and appropriate adjustments computed and executed, with a minimum time lag around 200 ms [19]. Grasping is also frequently time critical – an object can potentially move during a reach, or the initial contact by one finger can impart an impulse which will move the object if not quickly counteracted by the opposing finger. In addition, in many tasks if force-closure is not met at first contact, there may not be a second chance for grasping the objects. Hence, good initial contacts minimize natural costs associated with time and energy expended during corrective movements and reduce the chance of negative results.

The probability of achieving force-closure grasping is not the only criterion used to plan grasping movements, and our results showed that participants did not fully optimize this measure. Grasp planning may attempt to optimize other cost criteria in addition to force-closure grasping at first contact. Possible biomechanical cost functions, such as energy expenditure [29], joint mobility [30], muscle tension changes [31], mean torque changes [32], mean square rate of change of acceleration [33] and peak work [34] create competing criteria and constraints that must be simultaneously satisfied (see also [18]). Hence, perfect compensation may have been difficult to achieve for some covariance angles and the additional energy required may not be worth the small gain in grasping performance [35]. Especially, participants may sacrifice perfect compensation to increase comfort of their grasp [36]. Another possibility is that trajectories are selected to minimize the uncertainty in hand and finger positions [37]. Because participants grasped the object while it was out of view, this kind of uncertainty may have been non- negligible. However, in Fig. 1B, we showed that simulated errors in hand and finger position actually narrow the ranges of approaches that produce high force-closure probabilities, and reduce the probability of producing force-closure grasping.

It is important to note that our analysis of optimal grasping behavior with position uncertainty will not hold in all contexts. In the optimal analysis we permitted differences between the thumb and index finger contact times. This is appropriate for our experiment because the cylindrical object was held in a cradle. In general, time differences will not affect sufficiently heavy objects. For objects light enough to be toppled by contacting with one of the fingers, there will be an advantage to contacting the object with both fingers simultaneously. For this class of objects, there is a trade-off between minimizing the chance of knocking over the object and maximizing the chance of contacting the object. For instance, catching a frisbee by opening the fingers wider increases the chance to contact the object, but decreases the chance to catch it by contacting with both fingers simultaneously. We can extend the current analysis using similar Statistical Decision Theory principles and adding a new cost to penalize non-simultaneous contact of both fingers with the object.

An interesting question is what cues drive the compensation strategies we observed. Like previous studies, that showed integration of visual and proprioceptive information in motor tasks [38]–[40], participants may use visual and/or haptic feedback from the finger-cylinder contact to compensate for the position uncertainty of the object. Comparing fixed-end location and random-end location conditions suggests that the haptic error participants experienced in the latter condition is critical for compensating approach direction. Although participants observed the distribution of object position in both fixed-end location and random-end location conditions, only when they felt the location variability did they modify their approach direction. Nevertheless, the visual movement is not without effect. Particularly, we found gripwidth varies between the fixed-end location and random-end location conditions for most of the participants (excluding Participant 5). The results suggest that the sensorimotor system cannot ignore the cylinder motion even when it is uninformative. However, we found that participants did not adjust the approach direction with the covariance angle on the fixed-end location condition, but rather had a preferred approach direction of about 70 deg for all covariance angles.

We also examined whether there was evidence of learning by dividing trajectories into “early” and “late” groups and compared their characteristics. We did not find any significant differences in trajectory characteristics between the two groups, for any attempted split. The absence of significant learning effects is likely due to trajectory variability and the number of trials (100). However, it may also indicate that uncertainty compensation strategies are relatively constant.

In conclusion, the results show that people plan for the effects of uncertainty in selecting object contacts in purposive movement tasks.

## Materials and Methods

### Participants

Five right-handed (25–30 years old, 4 men and 1 woman) participants with normal or corrected-to-normal vision participated in the study for monetary compensation. The appropriate institutional review board approved the study protocol and informed consent was obtained based on the Declaration of Helsinki.

### Apparatus

Participants were instructed to reach rapidly with and then use a precision grasp to lift a cylindrical object (2.2 cm diameter and 11.5 cm height) held in a cradle on a platform mounted on a robotic arm that precisely moved the object (<1 mm error), Fig. 2A. Trajectory data were recorded by placing three infrared sensors on hard foam blocks attached to the fingernails of both index finger and thumb, which were tracked via an Optotrak 3020 with sampling rate 100 Hz. Reaches began with index finger and thumb placed on a reference block located 52 cm away from the average position of the cylinder and 1 cm above the platform plane. For convenience, we transformed all data to a coordinate frame in which the x-dimension corresponds to a straight line connecting the midpoint of the finger starting location to the midpoint of the average of the cylinder contact locations. The y-dimension is formed from the cross-product of the x-dimension and the cylinder's main axis. Finally, the z-dimension is perpendicular to x and y-dimension. Head-stabilized (via chinrest) participants viewed the cylinder at a distance of 52 cm through liquid crystal glasses which were used to occlude the object during reach time. Ear plugs and closed ear headphones were used to eliminate auditory cues to the motion of the object while it was out of view. A trip switch guaranteed the object was lifted at least 5 mm.

### Experimental paradigm

Participants selected a comfortable reference position by placing both fingers along the top edges of the reference block at the beginning of the experiment. At the start of each trial, participants viewed the cylinder for a period of time that depended on condition, and then vision was removed by shutting down the crystal liquid glasses. Thereafter, they were cued to rapidly reach, precision grasp and lift the object within 1200 ms while it was out of view. The trial was considered successful if the participant lifted the cylinder to trigger the switch within the timeout, however, none of the participants failed to grasp the cylinder within 1200 ms. The fingers had to be returned within 3 mm of their starting positions before the next trial was begun.

Participants were familiarized with the task by running a number of training trials on the non-motion condition. Once they were ready and felt comfortable with grasping the cylinder, the real trials began. On the no-motion condition the cylinder was stationary and the view time was 1 sec before glasses occluded the vision of the object. In the fixed-end location condition, the robotic arm moved the cylinder over a sequence of 5 random positions (1 sec each) sampled from a strongly oriented 2D Gaussian distribution. The cylinder was returned to the based (initial) position and the view was occluded after 1 sec. Thereafter, participants reached and grasped the cylinder, while it was out of view. Note that participants were told that the cylinder always returned to the base position. In the random-end location condition, the cylinder moved over a sequence of 5 positions (1 sec each) randomly generated by a strongly oriented 2D Gaussian distribution. Thereafter, the view of the object was occluded and the object moved to a new random position selected from the same Gaussian distribution. Finally, participants reached and grasped the cylinder, while it was out of view. In this condition, participants were told that the cylinder moved to a new position from within the same distribution of the 5 visible positions. For both fixed-end location and random-end location conditions, we used an 80 deg range of distribution orientations (−20°, 0°, 20°, 40°, 60°), designed to fit within, but strongly challenge participants' natural biomechanical reaching posture. The standard deviations of the major and minor axes of the covariance were 12 mm and 0.25 mm, respectively. Note that the covariance angle was defined with respect to the x-dimension of the coordinate frame. Trials from each covariance angle were blocked, and 100 trials of reaching, grasping and lifting the cylindrical object were performed for each block.

### Spatial trajectory data

Kernel density estimation was used to analyze the spatial distribution, velocities and orientations of the thumb and index finger trajectories as illustrated Fig. 2B. We computed the frequency that reach trajectories passed through a grid of spatial locations (1 mm×1 mm) in the 2D space (ignoring z-axis). We produced a density estimate from the frequency data using a Gaussian Kernel with standard deviation of 5.5 mm. The colors of the density map describe the probability density values with red corresponding to high and blue corresponding to low density regions. Smoothed estimates of the average velocity (arrows) and orientation (line segments) at each spatial location were also computed, because the number of measurements varied across cells. For each cell, velocity and orientation estimates were generated by performing a weighted average of these values from trajectories across neighboring cells, using a Gaussian filter with standard deviation of 3.5 mm as a weighting function.

### Trajectory analysis

We computed approach direction for the average trajectory for each participant on the two conditions (fixed and random-end location). Because averages are strongly affected by outliers, we excluded trajectories that had substantially different temporal characteristics. Note that trajectory data were spatially variable, but timing was consistent for trajectories with similar velocity profile. From the histogram of the time of maximal x-velocities, we selected the trajectories that fell within 80% the histogram mean (i.e., 10%–90% percentiles of the distribution) and averaged them. From the average trajectory, we computed the approach direction of the main axis of the ellipse that describes the spatial variation of the fingers centroids, Fig. 3A. The approach direction was computed as the direction of the principal axis of the covariance ellipse that describes the spatial variation of the sensor centroids across both trajectories and time points from the contact time through the preceding 100 ms (10 time steps). This 100 ms time period was selected because it corresponds to the average duration for closing the fingers (kinematically identified as the period in which the x-velocity is near zero and the y-velocity indicates the fingers are moving toward each other). Bootstrap resampling was used to estimate the mean and the standard error of the approach direction for each participant and covariance orientation. The mean and the standard error of the approach direction were computed from 100 bootstrap resamples. Because contact times were difficult to automatically detect from kinematic data, all candidate contact times were cross-checked both by visual inspection and by verifying that the index finger x- and y-velocity were near-zero and the distance of the finger to the cylinder was consistent with contact.

In addition, we measured MGA because it serves as a measure of position uncertainty [26] and changes with viewing eccentricity [27]–[28] and without vision [26]. In particular, fingers are opened wider for grasping objects without precise information about their position, most likely to avoid finger-object collision or missing the object. On each block of the experiment, we computed the average MGA by measuring the maximum distance between the centroid of sensors on the index finger and the thumb for every trajectory and averaging these values across the 100 trajectories. Note that this distance is 4.5 cm larger than between the contact surfaces of the index finger and thumb, due to the widths of the fingers and the 1 cm thick foam blocks the sensors were mounted on.

### Calibration of finger contact surfaces

To evaluate object-finger contact, we computed an estimate of the index finger and thumb contact surfaces relative to the sensor locations via a calibration procedure. The index finger and thumb were placed in grooves on a calibration block and the sensor locations were recorded. The orientation and position of the calibration block were also recorded by placing sensors in known locations on the calibration block. The groove angle, length and depth were precisely known due to the geometry of the block and the block required precision grip contacts similar to those made on the cylindrical object. We converted this information to approximate the index finger and thumb contact surfaces and computed a homogeneous transformation that converted between sensor and finger coordinates. In particular, finger coordinates had an origin at the center of the estimated region of potential contact, and had directions aligned so that one axis (the surface normal) was perpendicular to the calibration block surface (and hence index finger and thumb normals were parallel but opposite in direction), one axis pointed in the direction of the groove (along the length of the index finger and thumb), and the other axis roughly corresponded to finger width. Once projected into the x-y plane the surface approximation became a line segment that has a 1–2 mm error from the actual index finger/thumb contact surface.

### Evaluation of human strategies for grasping objects with directional position uncertainty

According to an optimal statistical modeling approach, the goal is to select a visuomotor grasping strategy that chooses a desired movement trajectory by optimizing the expected gain. The gain function takes into account the costs and benefits of possible outcomes of the movement [18]. Although there are multiple factors that affect grasping as described in the discussion, we focus on the gain provided by achieving force-closure at first contact. We use “optimal strategy” to refer to a grasping strategy that maximizes the probability of satisfying force-closure without post-contact adjustments. Selecting the optimal strategy can be described as an optimization problem as follows, where uppercase refers to random variables and lower case to instances.

The optimal movement policy 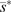 maximizes Eq. (1).
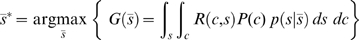
(1)where *S* is a random variable denoting an executed movement strategy, 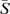 are reach plans, *C* represents cylinder locations, 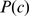 is the distribution of cylinder locations, 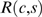 is an indicator function for the event of successful cylinder contact satisfying force-closure conditions given that the actual movement trajectory is *s*. The conditional distribution 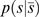 is the probability of performing the actual movement trajectory given that the planned movement trajectory is 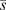. Finally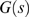 corresponds to the probability that force-closure conditions are satisfied following the movement trajectory *s*. In the “Text S1” we show how to solve Eq. (1) to find the optimal strategy.

To keep the analysis tractable, we made the following simplifications. Due to the shapes of both cylinder and fingers, and the post-contact lift direction of the cylinder we can safely neglect the spatial dimension along the cylinder's z-axis and focus on the perpendicular plane. Within the plane, the contact surfaces of the index finger and thumb can be approximated as line segments (see lower inset of Fig. 1A). The index finger's contact surface is parameterized by the line segment's midpoint, **r**_f_, directions 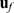 parallel and 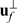 perpendicular to the surface, and a half-width 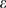. The thumb's contact surface is parameterized similarly, but with subscripts *th*. With this representation, a reach plan is a desired trajectory *S*(t) = {**r**_f_, **r**_th_, **u**_f_, **u**_th_}, where time dependence is suppressed inside the brackets to make the notation less complex.

Possible locations for the cylinder (with radius *ρ*) centroid is modeled as a random vector **c**_i_ sampled from a 2D Gaussian density *P*(*c*). To simplify the conditions for contact, we project the samples **c**_i_ into a frame of reference defined by the index finger's (or thumb's) contact surface, forming *contact coordinates*. Contact coordinates are simply the position of points in the environment with respect to the index finger and thumb surfaces. Let 
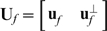
, then the contact coordinates for the cylinder with respect to the index finger are given by:
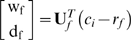



Contact coordinates for cylinder with respect to the thumb are similarly defined. The analysis of optimal grasping is presented in lower inset of Fig. 1A.

The force-closure indicator function 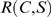, is based on the following necessary conditions for object-finger contacts to be in force-closure:

1. The index finger and thumb are touching the cylinder on the appropriate sides: 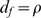 and 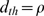

2. The contact point is within the width of the index finger or thumb: 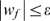, and 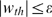

3. Nguyen [15] showed that a necessary condition for force-closure requires the index finger and thumb contacts to be at surface locations that are within each other's friction cones and include the center of mass (see upper inset of Fig. 1A). For a coefficient of static friction *μ* and given 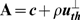, 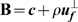 are the contact points of the thumb and index finger respectively, the necessary conditions for force-closure are:
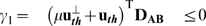
(2a)

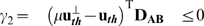
(2b)

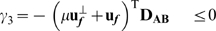
(2c)

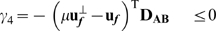
(2d)Where 
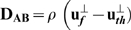
 is the vector between the contact points A, B.

Due to the cylinder geometry, the 4 conditions above are equivalent to the simplified condition, Eq. (3) that the angle between the surface normals is greater than 90 deg, for a coefficient of fiction *μ* = 1 (reasonable for skin-plastic contact):
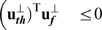
(3)


Hence, the indicator function 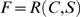 is given by Eq. 4:
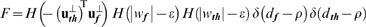
(4)Where *H* and *δ* denote Heaviside step function and the Delta Dirac, respectively (see Text S1 for more details)

To compare human performance to optimal, we estimated the probability of force-closure from trajectory data. For each participant and condition, we treated the set of trajectories as samples from 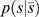, the distribution of trajectories given the participant's strategy in that condition. To estimate 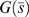, we also computed the expected probability of force-closure for each trajectory and then averaged across trajectories, which can be considered a kind of Monte Carlo integration.

We estimated the expected probability of force-closure 
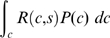
 associated with an observed trajectory by measuring the proportion of sampled cylinders that could have been contacted by the fingers along the trajectory and that would have satisfied the conditions for force-closure, had the cylinder been at one of these sampled locations. For each movement trajectory in a random-end location condition with a specific covariance angle, we generated *M* (where *M* = 1000) virtual “cylinder positions” drawn from a 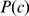 with mean and covariance equal to those used in the random-end location condition block of the experiment. The main difficulty in computing this estimate was that either the index finger or thumb was stopping when it made contact with the actual cylinder before sweeping through all sampled cylinder locations. After one of the fingers made contact, the remaining trajectory was biased by the knowledge of the actual cylinder location, and thus should not be used to estimate the probability of force-closure given the reach plan. However, evaluating the conditions for force-closure requires both fingers to contact the cylinder. To overcome this problem, we took advantage of the fact that trajectories were near-linear and had low-variability close to the center of the cylinder distribution. Thus, we assumed that the remaining trajectory of the non-contacting finger can be replaced by extrapolating in the direction of average trajectory (across all trials) for that finger at the contact time, until the extrapolation either contacted or missed the cylinder. Because the proportion of locations intersected was affected by this collision, we normalized the counts by the number of cylinders that the ideal strategy would successfully grasp if the index finger and thumb were stopped at the same locations. This computation is illustrated for two different trajectories in Figs. 4A, and 4B, respectively.

We tested the optimality of participants' grasp strategies and to provide a comparison, we also estimated the deterioration in grasping performance if participants do not compensate for uncertainty. To provide a baseline comparison for this grasp performance measure, we estimated the probability of force-closure if participants had adopted a non-compensation strategy for the random-end location condition (and hence would not have compensated for the cylinder location uncertainty). We simulated the non-compensation strategy by estimating the expected probability of force-closure for no-motion condition trajectories on the random-end location condition cylinder locations.

## Supporting Information

Text S1 Supplementary Materials
(0.09 MB DOC)
