# Supplementary material for: Grasping Objects with Environmentally Induced Position Uncertainty
Source: PLoS Comput Biol. 2009 Oct 16;5(10):e1000538. doi: 10.1371/journal.pcbi.1000538 (PMC2756623; doi:10.1371/journal.pcbi.1000538)
Supplement: Text S1 — Supplementary Materials (0.09 MB DOC) [file pcbi.1000538.s001.doc]

**Supplementary Materials**

In the methods section we outlined a Bayesian decision theoretic analysis of grasping under position uncertainty, which optimizes reach plans $\bar{S}$ over a function $R\left(C,S\right)$ that rewards trajectories with a high probability of achieving force-closure.

$$\bar{s}^{*}=\mathop{\mathrm{argmax}}\limits_{\bar{s}} \left\{ G\left(\bar{s}\right)=\int_{s} \int_{c} R\left(c,s\right)P(c) p\left(s|\bar{s}\right) ds dc\right\}$$

In this section we show how to optimize the probability of force-closure for a trajectory $G\left(\bar{s}\right)()$ over reach plans. In particular, we prove that the approach that maximizes the probability of these contact conditions is aligned with the principal axis of the covariance of the cylinder distribution.

From the contact conditions described in the methods section, the contact indicator $R\left(C,S\right)$ has the form:

$$F=H\left(-\left(u_{th}^{\perp}\right)^{T}u_{f}^{\perp}\right) H\left(\left|w_{f}\right|-\varepsilon\right) H\left(\left|w_{th}\right|-\varepsilon\right) \delta\left(d_{f}-\rho\right) \delta\left(d_{th}-\rho\right)$$

Where *H* and *δ* denote Heaviside step function and the Delta Dirac, respectively.

To compute the probability that a trajectory will produce a force-closure grasp (*F*=1), we need to compute the integral across *C,* which determines the fraction of possible cylinder locations that intersect the trajectory and satisfy the conditions above. Because the cylinder is in a cradle, the index finger and thumb can contact at different times and will not topple the cylinder. Thus, we can ignore the relative timing and focus on the spatial paths of the trajectories.

The integral$\int_{c} R\left(c,s\right)p\left(c\right) dc$involves integrating a Gaussian distribution times a Dirac Delta over the contact coordinates that depend linearly on *C,* which results in another Gaussian distribution expressed in contact coordinates. It is easier to use the properties of Gaussian random variables under linear transformations to express this result. Thus we proceed by using the distribution of cylinder locations to induce a probability distribution on contact conditions. For convenience, we put the origin of trajectory space at the mean of the cylinder distribution. The density of cylinder locations is Gaussian with zero mean and covariance $\Sigma=V\left[\begin{matrix} \sigma_{\min}^{2} & 0 \\ 0 & \sigma_{\max}^{2} \end{matrix}\right]V^{T}$where$V=\left[\begin{matrix} \mathbf{v}_{m}^{\perp} & \mathbf{v}_{m} \end{matrix}\right]$ is an orthonormal matrix composed of a unit vector $\mathbf{v}_{m}$ in the direction of maximal variance and its orthogonal complement. We assume that the maximal variance $\sigma_{\max}^{2}$ is much larger than$\sigma_{\min}^{2}$, making the covariance strongly oriented.

The integral $\int_{c} R\left(c,s\right)p\left(c\right) dc$ simplifies if we rewrite the contact coordinates of one finger in terms of the other. In particular, $\left[w_{th},d_{th}\right]$ is written in terms of $\left[w_{f},d_{f}\right]$ as follows:

$$\left[\begin{matrix} w_{th} \\ d_{th} \end{matrix}\right]=\mathbf{U}_{th}^{T}\left(\mathbf{U}_{f}\left[\begin{matrix} w_{f} \\ d_{f} \end{matrix}\right]+r_{f}-r_{th}\right)$$

Because linear transformations of Gaussian random variables remain Gaussian, the distribution on cylinder locations$P\left(\vec{c}\right)=N\left(\vec{0},\Sigma\right)$, induces a distribution on index finger contact coordinates, conditioned on index finger trajectory positions.

$$P\left(w_{f},d_{f}|r_{f}\right)=N\left(-\mathbf{U}_{f}^{T}r_{f},\mathbf{U}_{f}\Sigma\mathbf{U}_{f}^{T}\right)$$

Given this induced distribution, it is relatively straightforward to compute the probability that the force-closure conditions hold. We must evaluate the cumulative probability that the contact conditions hold across a path$\left\{r_{f}(t),r_{th}(t)\right\}$. However, we need to compute the probability that *F*=1 at$\left\{r_{f}(t),r_{th}(t)\right\}$, a single time point *t* in the trajectory:

$$P\left(\left.F=1\right| r_{f}\left(t\right),r_{th}\left(t\right)\right)=H\left(-\left(u_{f}^{\perp}\right)^{T}u_{th}^{\perp}\right)\int_{-\varepsilon}^{\varepsilon} P\left(w_{f},d_{f}=\rho|r_{f}\right)P\left(w_{th},d_{th}=\rho|r_{th}\left(t\right),r_{f}\left(t\right),w_{f}\right) dw_{f}$$

Where $P\left(w_{th},d_{th}|r_{th},r_{f},w_{f}\right)=\delta\left(\left[\begin{matrix} w_{th} \\ d_{th} \end{matrix}\right]-\mathbf{U}_{th}^{T}\left(\mathbf{U}_{f}\left[\begin{matrix} w_{f} \\ d_{f} \end{matrix}\right]+r_{f}-r_{th}\right)\right)$

The total probability of collision is the integral of $P\left(\left.F=1\right| r_{f}\left(t\right),r_{th}\left(t\right)\right)$ over the trajectory. However, to compute the probability across a trajectory, we assume that if the index finger or thumb collides with the cylinder during the trajectory, it stops while the other finger continues on its same path. Although unrealistic for large movements, for our task the gap between the object and the non-contacting finger is never more than a few cm, making path changes due to propagation of the collision impulse through the hand less of an issue. This assumption allows us to decouple the trajectories of the index finger and the thumb and integrate them separately.

$$P\left(\left.F=1\right|S\right)=\iint_{t_{f},t_{th}} P\left(\left.F=1\right| r_{f}\left(t_{f}\right),r_{th}\left(t_{th}\right)\right) dt_{f} dt_{th}$$

The trajectory that maximizes this expression is optimal in the sense that it has the best chance (on average) to achieve force-closure at contact. To maximize this expression, we take advantage of the following geometric argument. As the index finger and thumb sweep toward each other, they create an overlap area that represents the space of possible cylinders that can be intersected. The maximal overlap occurs when the finger contact surfaces are perpendicular to each other and the surface centroids move along a common path. Moreover, the best path is a straight line, because non-straight paths introduce spatial positions where the one finger might intersect a cylinder but the other could miss it. In addition, when the fingers move toward each other along straight line path, any cylinder contacted will satisfy all the force-closure constraints. Thus, the problem reduces to find a straight line path that maximizes the integral above. We can parameterize the path as a straight line perpendicular to the contact surface $r=\left[\begin{matrix} 0 \\ \tau\end{matrix}\right]$. Here *τ* represents a path parameter that carries the finger through the origin of the space. The direction of this path through the cylinder distribution is determined by the finger surface normal since we define contact coordinates relative to the index finger/thumb orientation. Because along these paths the contact conditions are guaranteed to be satisfied, the probability of force-closure for a path reduces to a 2D integral over the cylinder distribution.

$$P\left(\left.F=1\right| r_{f}\left(t\right),r_{th}\left(t\right)\right)=\int_{-T}^{T} \int_{-\varepsilon}^{\varepsilon} N\left(-\mathbf{U}_{f}^{T}\left[\begin{matrix} 0 \\ \tau\end{matrix}\right],\mathbf{U}_{f}V\left[\begin{matrix} \sigma_{\min}^{2} & 0 \\ 0 & \sigma_{\max}^{2} \end{matrix}\right]V^{T}\mathbf{U}_{f}^{T}\right)dd_{f}d\tau$$

where *T* is the maximum distance of the finger from the origin, and the integration is symmetric due to the exchangeability of index finger and thumb paths. Assuming that the maximum gripwidth 2*T* for the ideal agent is larger than the finger’s contact surface width 2$\varepsilon$, the integral is maximized when$\mathbf{U}_{f}=V^{T}$, which is when the longer integration region is parallel to the principle axis of the covariance matrix. Moreover, gripwidth should be made larger than fingerwidth - if biomechanically possible - because gripwidth is a controlled variable. In addition, the optimal strategy sets$p\left(S|\bar{S}\right)$ equal to a Dirac Delta since any variability in path can only decrease this integral. In summary, the optimal strategy involves the index finger and thumb approaching the cylinder distribution center along a straight line path aligned with the principle axis of the cylinder distribution’s covariance matrix.
